# Relevant factors for the optimal duration of extended endocrine therapy in early breast cancer

**DOI:** 10.1007/s10549-017-4601-1

**Published:** 2017-12-12

**Authors:** Erik J. Blok, Judith R. Kroep, Elma Meershoek-Klein Kranenbarg, Marjolijn Duijm-de Carpentier, Hein Putter, Gerrit-Jan Liefers, Johan W. R. Nortier, Emiel J. Th. Rutgers, Caroline M. Seynaeve, Cornelis J. H. van de Velde, On Behalf of the IDEAL Study Group

**Affiliations:** 1 Department of Surgery, Leiden University Medical Center, 2300 RC Leiden, The Netherlands; 2 Department of Medical Oncology, Leiden University Medical Center, P.O. Box 9600, Leiden, The Netherlands; 3 Department of Medical Statistics, Leiden University Medical Center, Leiden, The Netherlands; 4 Department of Surgery, Netherlands Cancer Institute, Amsterdam, The Netherlands; 5 Department of Medical Oncology, Erasmus MC Cancer Institute, Rotterdam, The Netherlands

**Keywords:** Letrozole, Extended, Adjuvant, Subgroup, Postmenopausal, IDEAL

## Abstract

**Purpose:**

For postmenopausal patients with hormone receptor-positive early breast cancer, the optimal subgroup and duration of extended endocrine therapy is not clear yet. The aim of this study using the IDEAL patient cohort was to identify a subgroup for which longer (5 years) extended therapy is beneficial over shorter (2.5 years) extended endocrine therapy.

**Methods:**

In the IDEAL trial, 1824 patients who completed 5 years of adjuvant endocrine therapy (either 5 years of tamoxifen (12%), 5 years of an AI (29%), or a sequential strategy of both (59%)) were randomized between either 2.5 or 5 years of extended letrozole. For each prior therapy subgroup, the value of longer therapy was assessed for both node-negative and node-positive patients using Kaplan Meier and Cox regression survival analyses.

**Results:**

In node-positive patients, there was a significant benefit of 5 years (over 2.5 years) of extended therapy (disease-free survival (DFS) HR 0.67, *p* = 0.03, 95% CI 0.47–0.96). This effect was only observed in patients who were treated initially with a sequential scheme (DFS HR 0.60, *p* = 0.03, 95% CI 0.38–0.95). In all other subgroups, there was no significant benefit of longer extended therapy. Similar results were found in patients who were randomized for their initial adjuvant therapy in the TEAM trial (DFS HR 0.37, *p* = 0.07, 95% CI 0.13–1.06), although this additional analysis was underpowered for definite conclusions.

**Conclusions:**

This study suggests that node-positive patients could benefit from longer extended endocrine therapy, although this effect appears isolated to patients treated with sequential endocrine therapy during the first 5 years and needs validation and long-term follow-up.

## Introduction

In hormone receptor-positive (HR+) breast cancer, adjuvant endocrine therapy is used to decrease the risk for recurrence, and improve the overall survival (OS). Where tamoxifen for 5 years has been the standard adjuvant endocrine therapy for a long period of time, currently, treatment regimens for adjuvant endocrine therapy are mostly based on 5 years of an aromatase inhibitor (AI), or a sequential strategy of tamoxifen followed by an AI. Among others, the Tamoxifen Exemestane Adjuvant Multinational (TEAM) trial showed that after 5 and 10 years of follow-up, there was no difference in disease-free survival (DFS) between patients randomized to either tamoxifen followed by exemestane or exemestane monotherapy [1, 2]. These results were confirmed in a meta-analysis performed by the Early Breast Cancer Trialists Collaborative Group (EBCTCG) [3].

Despite the value of adjuvant endocrine therapy, it is known that the risk for recurrence in HR+ breast cancer remains linear up to at least 15 years after diagnosis prompting to study the value of extended endocrine therapy. After 5 years of tamoxifen, it has been established that extended therapy beyond 5 years leads to a modest reduction in recurrences, but not in overall survival [4, 5]. This has been particularly observed for patients with node-positive disease [6].

The value of extended endocrine therapy after a 5 years regimen including an AI (either upfront or after 2–3 years of tamoxifen) is less clear. Recently, three independent studies did not show a significant benefit of (longer) extended endocrine therapy for the total study population [7–9]. In the NSABP B-42 trial, patients who earlier received either 5 years of an AI, or a sequential treatment of tamoxifen followed by an AI until 5 years, were randomized between 5 years of extended letrozole or placebo. After 5 years, there was no significant benefit of 5 years of letrozole over placebo. In the subgroup analysis, however, a significant benefit for patients who received prior tamoxifen followed by an AI (HR 0.75, *p* = 0.04) was found, which was not observed in patients who were treated upfront with AI monotherapy for 5 years (HR 0.91, *p* = 0.34) [7].

In the Dutch ‘Investigation on the Duration of Extended Adjuvant Letrozole treatment’ (IDEAL) trial, 1824 postmenopausal patients, who received any form of primary adjuvant endocrine therapy for 5 years, were randomized between extended letrozole for 2.5 or 5 years. The results of this trial were published recently by our group, and identified no subgroup that benefitted significantly from 5 instead of 2.5 years of extended therapy [9]. In the IDEAL trial, approximately 60% was treated initially with the sequential scheme, whereas 30% was treated with an upfront aromatase inhibitor only, and approximately 10% was treated with tamoxifen monotherapy.

In the Dutch study on ‘Duration of Anastrozole therapy after 2–3 years Tamoxifen as Adjuvant therapy’ (DATA), postmenopausal patients were randomized after 2–3 years of tamoxifen between 3 years of anastrozole (standard arm, duration of endocrine therapy 5–6 years in total) or 6 years of anastrozole (extended duration, 8–9 years in total). Also in this trial, no effect of extended AI (anastrozole) therapy was shown for the total population. However, this study did observe a significant benefit of longer AI therapy in high-risk subgroups, in particular, in patients with lymph-node-positive disease [8].

Combining the conclusions on the subgroup analyses of the NSABP B-42 and DATA trials, it is suggested that extended therapy might be the most beneficial for node-positive patients who were previously treated with tamoxifen followed by an AI. However, the optimal duration of extended therapy is not clear, since the regimens and populations in both trials differ too much for direct comparisons. In view of the above-mentioned data, we performed an additional subgroup analysis in the IDEAL trial. The aims of the current subanalyses were to investigate the effects of primary adjuvant treatment and nodal status on the optimal duration of extended adjuvant endocrine therapy. Furthermore, similar analyses were conducted in the subgroup of patients that previously participated in the TEAM trial, as this subgroup was randomized for the initial therapy.

## Methods

### IDEAL trial cohort

In the phase-3 IDEAL trial, 1824 postmenopausal patients were randomized at diagnosis between 2.5 or 5 years of letrozole, after 5 years of any type of adjuvant endocrine therapy for early HR+ breast cancer. Patients needed to be disease free at the moment of randomization. Furthermore, a maximum of 2 years was allowed between finishing earlier endocrine therapy and starting extended therapy. As the treatment arms during the first 2.5 years were equal, no differences can be expected during this period. Therefore, for the current analysis, patients that encountered an event or stopped therapy during the first 2.5 years were excluded, and the survival analysis started at 2.5 years after randomization at which time point the treatment arms diverge. Details of the trial, data collected, and the primary results have recently been reported elsewhere [9, 10].

A total of 438 IDEAL patients (24%) also participated in the TEAM trial during the first 5 years of their adjuvant endocrine therapy. In this phase-III study, postmenopausal patients with early HR+ breast cancer were randomized at diagnosis between 5 years of exemestane, or 2.5 years of tamoxifen followed by 2.5 years of exemestane (sequential scheme). In case they were disease free and finished 5 years of therapy, and their hospital participated in the IDEAL trial, they were eligible for inclusion in the IDEAL trial. In order to correct for a possible allocation bias in the distribution of previous endocrine therapy between node-negative and node-positive patients, all analyses were repeated in the cohort of patients that participated in the TEAM trial as these patients were not subjected to allocation bias due to the randomization already at primary diagnosis.

The IDEAL trial is registered in the Netherlands with the Netherlands Trial Register, NTR3077, the Dutch Breast Cancer Research Group (BOOG 2006-05) and Eudra-CT 2006-003958-16. The original study was conducted in compliance with the guidelines of the Declaration of Helsinki, International Conference on Harmonisation and Good Clinical Practice.

### Endpoints

The primary endpoint of the IDEAL trial was disease-free survival (DFS) defined as the time from randomization to recurrence (either local, regional, or distant), new primary breast tumors (DCIS or invasive) or death due to any cause. For the current analysis, DFS was also the primary study endpoint, with follow-up starting at 2.5 years after randomization with a 10% margin. The secondary outcomes for this analysis were overall survival (OS), defined as time to death due to any cause starting at 2.5 years after randomization, and distant metastasis-free interval (DMFi), defined as time to distant recurrence starting at 2.5 years after randomization.

### Statistical analysis

The analyses for primary and secondary outcomes (DFS, OS, and DMFi) of the current study were performed using Kaplan–Meier analysis, stratified for the type of endocrine therapy during the first 5 years, and nodal status at diagnosis. Hazard ratios (HRs) and treatment-by-marker interactions were estimated using Cox regression analysis.

## Results

### Cohorts

Of the 1824 postmenopausal patients enrolled in the IDEAL trial, 1339 were disease free and on letrozole therapy at 2.5 years after randomization, and were eligible for the current analysis. There were no significant differences in patient baseline characteristics between the randomized treatment arms in this subcohort (Table 1).

**Table 1:** Characteristics of the IDEAL study cohort of patients who were disease free and on therapy after 2.5 years of extended treatment

	2.5 years		5 years	
	*N*	%	*N*	%
Age at randomisation				
< 55 years	191	28.6	197	29.4
55–65 years	288	43.0	283	42.2
65–75 years	151	22.6	136	20.3
> 75 years	39	5.8	54	8.1
Nodal status				
pN0/pN0(i+)	176	26.3	171	25.5
pN1(mi)/N1/N2/N3	493	73.7	499	74.5
Tumor type				
Ductal	508	75.9	547	81.6
Mucinous	5	0.7	6	0.9
Medullar	1	0.1	2	0.3
Lobular	113	16.9	87	13.0
Other/unknown	42	6.2	28	4.2
Histological grade				
Grade 1	115	17.2	102	15.2
Grade 2	278	41.6	281	41.9
Grade 3	205	30.6	217	32.4
Gx	71	10.6	70	10.4
Progesteron receptor status				
Negative	113	16.9	136	20.3
Positive ≥ 10%	528	78.9	510	76.1
HER2 status				
Negative	242	36.2	246	36.7
Positive	67	10.0	63	9.4
Unknown	360	53.8	309	53.9
Performed final surgery				
Breast conserving	335	50.1	324	48.4
Mastectomy	331	49.5	344	51.3
Prior chemotherapy				
No	212	31.7	198	29.6
Yes	457	68.3	472	70.4
Prior endocrine treatment				
5 years tamoxifen	76	11.4	78	11.6
5 years AI	177	26.5	192	28.7
2–3 years tam- > 3–2 years AI	416	62.2	400	59.7
Time after stop hormonal therapy (months)				
0– < 6	602	90.0	610	91.0
6– < 12	30	4.5	27	4.0
12–27	37	5.5	33	4.9

Of the 438 patients who also participated in the TEAM trial, 311 patients were disease free and on therapy at 2.5 years after randomization in the IDEAL study, and therefore eligible for our additional analysis. Patient characteristics of the IDEAL-only and IDEAL/TEAM patients are described in Table 2. Compared with the IDEAL-only cohort (not participating in TEAM), IDEAL/TEAM patients were significantly older at randomization, more often treated with breast conserving therapy (55% vs. 47.5%, *X* ^2^ *p* = 0.037) and less often treated with chemotherapy (42.1% vs. 77.6%) (Table 2).

**Table 2:** Characteristics of the IDEAL patients that participated earlier in the TEAM trial

	Participation in TEAM trial				
	No		Yes		*X* ^2^ *p* value
	*N*	%	*N*	%	
Age at randomisation					
< 55 years	380	37.0	8	2.6	< 0.001
55–65 years	445	43.3	126	40.5	
65–75 years	163	15.9	124	39.9	
> 75 years	40	3.9	53	17.0	
Nodal status					
pN0/pN0(i +)	273	26.6	74	23.8	0.33
pN1(mi)/N1/N2/N3	755	73.4	237	76.2	
Tumor type					
Ductal	803	78.1	252	81.0	0.84
Mucinous	9	0.9	2	0.6	
Medullar	2	0.2	1	0.3	
Lobular	160	15.6	40	12.9	
Other/unknown	54	5.3	16	5.1	
Histological grade					
Grade 1	161	15.7	56	18.0	0.06
Grade 2	422	41.1	137	44.1	
Grade 3	322	31.3	100	32.2	
Gx	123	12.0	18	5.8	
Progesteron receptor status					
Negative	179	17.4	70	22.5	0.19
Positive ≥ 10%	807	78.5	231	74.3	
HER2 status					
Negative	403	39.2	85	27.3	< 0.001
Positive	125	12.2	5	1.6	
Unknown	500	48.6	221	71.1	
Performed final surgery					
Breast conserving	488	47.5	171	55.0	0.04
Mastectomy	535	52.0	140	45.0	
Prior chemotherapy					
No	230	22.4	180	57.9	< 0.001
Yes	798	77.6	131	42.1	
Prior endocrine treatment					
5 years tamoxifen	150	14.6	4	1.3	< 0.001
5 years AI	206	20.0	163	52.4	
2–3 years tam- > 3–2 years AI	672	65.4	144	46.3	
Time after stop hormonal therapy (months)					
0– < 6	928	90.3	284	91.3	0.63
6– < 12	43	4.2	14	4.5	
12–27	57	5.5	13	4.2	

Regarding the prior endocrine therapy strategy, 816 IDEAL patients (60.9%) were treated with a sequential scheme of tamoxifen followed by an AI, 369 patients (27.6%) were treated with AI monotherapy, and 154 patients (11.5%) were treated with tamoxifen monotherapy. In the TEAM subgroup, 46.3% was treated with a sequential scheme, and 52.4% with AI monotherapy, as expected due to the TEAM trial design. Another four TEAM patients were treated with tamoxifen monotherapy because of refusal of switch to AI after 2.5 years of tamoxifen.

### Main subgroup analysis in all patients

In the total selected IDEAL patient group (*n* = 1339), 167 patients encountered a DFS event during follow-up (median follow-up of 7 years, including the first 2.5 years).

**Fig. 1:**
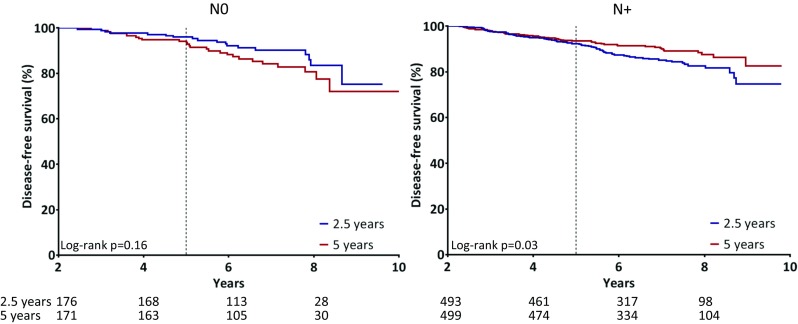
Kaplan–Meier analysis for disease-free survival of all patients that were disease free and on therapy after 2.5 years, stratified for nodal status. Log-rank tests were used to assess the differences between treatment arms for each subgroup (reported as *p* values)

For node-negative patients, no benefit of longer endocrine therapy was found (HR 1.53, *p* = 0.16, 95% CI 0.84–2.80). In contrast, for node-positive patients we observed a beneficial effect of longer extended therapy (HR 0.67, *p* = 0.03, 95% CI 0.47–0.96), with a HR for interaction between nodal subgroups of 0.44 (95% CI 0.22–0.88, *p* = 0.02) (Table 3, Fig. 1).

**Table 3:** A subgroup analysis for the effect of 5 versus 2.5 years of extended letrozole on disease-free survival (DFS), distant metastasis-free interval (DMFi) and overall survival, stratified on prior endocrine therapy and nodal status

	All patients	Events	HR	*p* value	95.0% CI	*p* for interaction	TEAM cohort	Events	HR	*p* value	95.0% CI	*p* for interaction
DFS												
All pretreatments	N0 (*n* = 347)	44	1.53	0.16	0.84–2.80	0.02	N0 (*n* = 74)	16	2.12	0.15	0.77–5.85	0.06
	N+ (*n* = 992)	123	0.67	0.03	0.47–0.96		N+ (*n* = 237)	35	0.64	0.20	0.33–1.26	
5 years tamoxifen	N0 (*n* = 48)	3	1.83	0.62	0.17–20.23	0.50	N0 (*n* = 0)	–	–	–	–	–
	N+ (*n* = 106)	11	0.87	0.81	0.26–2.85		N+ (*n* = 4)	0	–	–	–	
5 years AI	N0 (*n* = 102)	14	1.88	0.26	0.63–5.67	0.20	N0 (*n* = 38)	9	1.31	0.70	0.34–5.08	0.84
	N+ (*n* = 266)	35	0.81	0.52	0.41–1.57		N+ (*n* = 125)	19	1.09	0.85	0.44–2.69	
2–3 years tamoxifen > 3–2 years AI	N0 (*n* = 196)	27	1.44	0.35	0.67–3.07	0.05	N0 (*n* = 36)	7	3.68	0.12	0.71–18.97	0.02
	N+ (*n* = 620)	77	0.60	0.03	0.38–0.95		N+ (*n* = 108)	16	0.37	0.07	0.13–1.06	
DMFi												
All pretreatments	N0 (*n* = 347)	19	1.43	0.44	0.57–3.55	0.12	N0 (*n* = 74)	5	1.89	0.49	0.31–11.40	0.19
	N+ (*n* = 992)	67	0.63	0.06	0.38–1.03		N+ (*n* = 237)	15	0.41	0.11	0.14–1.22	
5 years tamoxifen	N0 (*n* = 48)	1	–	–	–	–	N0 (*n* = 0)	0	–	–	–	–
	N+ (*n* = 106)	6	1.10	0.91	0.22–5.45		N+ (*n* = 4)	0	–	–	–	
5 years AI	N0 (*n* = 102)	5	1.55	0.63	0.26–9.36	0.65	N0 (*n* = 38)	2	1.18	0.91	0.07–19.95	0.98
	N+ (*n* = 266)	16	0.98	0.96	0.37–2.60		N+ (*n* = 125)	8	1.01	0.99	0.25–4.04	
2–3 years tamoxifen > 3–2 years AI	N0 (*n* = 196)	13	1.32	0.62	0.44–3.93	0.14	N0 (*n* = 36)	3	2.56	0.44	0.23–28.30	0.09
	N+ (*n* = 620)	45	0.50	0.03	0.27–0.94		N+ (*n* = 108)	7	0.14	0.07	0.02–1.15	
OS												
All pretreatments	N0 (*n* = 347)	22	1.63	0.27	0.69–3.84	0.26	N0 (*n* = 74)	8	2.07	0.32	0.49–8.71	0.32
	N+ (*n* = 992)	70	0.89	0.61	0.55–1.42		N+ (*n* = 237)	24	0.88	0.76	0.40–1.97	
5 years tamoxifen	N0 (*n* = 48)	1	–	–	–	–	N0 (*n* = 0)	0	–	–	–	–
	N+ (*n* = 106)	8	1.80	0.42	0.43–7.55		N+ (*n* = 4)	0	–	–	–	
5 years AI	N0 (*n* = 102)	5	4.28	0.20	0.47–38.58	0.23	N0 (*n* = 38)	3	2.13	0.55	0.18–24.88	0.65
	N+ (*n* = 266)	20	0.96	0.92	0.40–2.30		N+ (*n* = 125)	13	1.14	0.82	0.38–3.39	
2–3 years tam– > 3–2y AI	N0 (*n* = 196)	16	1.23	0.69	0.46–3.29	0.45	N0 (*n* = 36)	5	2.11	0.41	0.35–12.66	0.34
	N+ (*n* = 620)	42	0.75	0.36	0.41–1.39		N+ (*n* = 108)	11	0.71	0.58	0.22–2.34

**Fig. 2:**
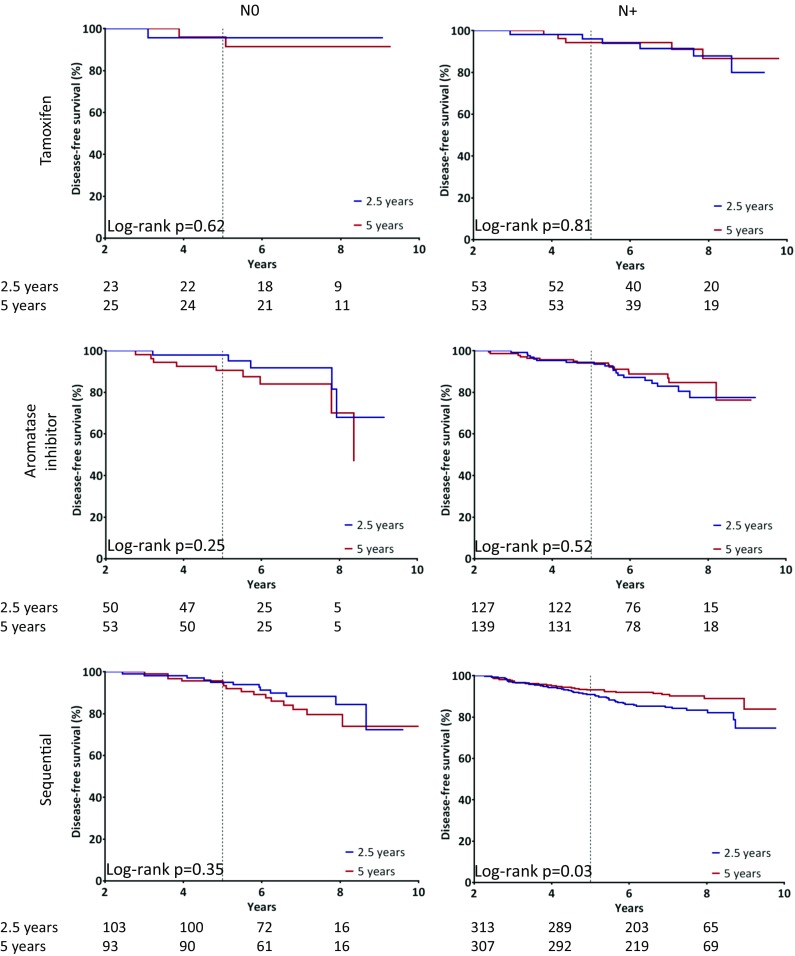
Kaplan–Meier analysis of the main analysis in all patients that were disease free and on therapy after 2.5 years. Results are shown for disease-free survival, for the subgroups stratified on prior endocrine therapy and nodal status. Log-rank tests were used to assess the differences between treatment arms for each subgroup (reported as *p* values)

When stratified for nodal status and type of endocrine therapy during the primary adjuvant therapy, we only observed the benefit of 5 years over 2.5 years of letrozole for node-positive patients in patients treated with prior sequential endocrine therapy (8 year DFS after randomization 89% vs. 83.4%, HR 0.61, *p* = 0.037, 95% CI 0.38–0.97) (Fig. 2). In this subgroup, the *p* value for the treatment by subgroup interaction test based on nodal status was 0.05, indicating a significantly higher treatment effect in node-positive compared to node-negative patients. In all other considered subgroups, no benefit of longer extended therapy was observed (Table 3).

For the endpoint DMFi, similar results were observed (Table 3). In node-positive patients previously treated with sequential therapy, a benefit of 5 over 2.5 years of letrozole was shown (HR 0.50, *p* = 0.03, 95% CI 0.27–0.94), but no differential effect between the treatment durations was observed for all other subgroups (*p* for interaction 0.14). For the endpoint OS, no benefit of longer extended therapy was shown for any of the subgroups (Table 3).

### Additional subgroup analysis in TEAM patients

With respect to the additional analysis in the TEAM patient subgroup (*n* = 311), 50 patients had a DFS event, of which 19 were DMFi events and 31 OS events. For DFS, a benefit of longer extended therapy was observed for node-positive patients pretreated with sequential therapy, however, without statistical significance (8 year DFS after randomization 90% vs. 76.1%, HR 0.37, *p* = 0.07, 95% CI 0.13–1.06). For DMFi, a similar nonsignificant benefit of longer therapy was found for the same subgroup (HR 0.14, *p* = 0.07, 95% CI 0.02–1.15). Regarding OS, no benefit was realized for any of the subgroups (Table 3).

## Discussion

In this analysis of IDEAL patients, we found a significant benefit of longer (5 vs. 2.5 years) extended letrozole therapy on disease-free and distant-metastasis-free survival, for node-positive patients, and in particular for those who received sequential adjuvant endocrine therapy during the first 5 years. In contrast, patients treated with AI monotherapy had no benefit of longer extended therapy, irrespective of nodal status. For overall survival, no significant benefit of longer extended letrozole was observed in any subgroup, although the follow-up is relatively short for definite conclusions hereon.

The distribution of patients pretreated with tamoxifen (followed by an AI) or with AI monotherapy in the full IDEAL cohort might have been subject to allocation bias. Therefore, we performed an additional analysis in the IDEAL patients who also participated in the TEAM trial. Using the randomization of the TEAM trial, we balanced the previous endocrine therapy subgroups for baseline characteristics. In this additional analysis, similar numerical results were observed, although without statistical significance. This is most likely explained by the lack of power due to the smaller population size, and the low number of events in general. However, the similarity between the HRs for the total IDEAL cohort and the TEAM subgroup indicates that the results from the IDEAL cohort are not explained by an allocation bias.

The observation that (longer) extended therapy was only of value for node-positive patients, being at higher risk of recurrent disease, is in line with previous observations. In a meta-analysis by Ibrahim et al., in which all patients were pretreated with tamoxifen monotherapy, a subgroup analysis showed that the positive effect of extended endocrine therapy on breast cancer recurrence was only observed in node-positive patients (OR 0.70, 95% CI 0.58–0.84), and not in node-negative patients (OR 0.96, 95% CI 0.71–1.29) [6]. Remarkably, in our analysis there was no benefit of longer extended therapy in either node-negative or node-positive patients that were treated with tamoxifen monotherapy. However, tamoxifen monotherapy for the first 5 years was not considered as standard therapy anymore during the conduct of the IDEAL trial, and most likely there might have been a selection bias of very-low-risk patients who remain on tamoxifen after 2–3 years instead of switching to an AI. In these low-risk patients, a benefit of extended therapy is unlikely. Furthermore, tamoxifen monotherapy as prior endocrine therapy was a very small subgroup (12%) in the IDEAL trial, leading to a lack of power for conclusions in this subgroup.

The results of our analysis suggest that when patients were pretreated with AI monotherapy for 5 years, there was no additional effect of 5 over 2.5 years of extended AI therapy. A possible explanation could be that the maximal treatment effect of aromatase inhibitors is reached after approximately 7.5 years. Therefore, after 5 years of AI monotherapy, an additional 5 years would have no benefit over 2.5 years. However, the results from this relative small subgroup analysis need to be interpreted with care, and should be validated in a meta-analytical setting before final conclusions can be drawn.

In all node-negative subgroups, there is a trend toward worse outcome for longer therapy, although none of these effects are statistically significant (Table 3). For overall survival, this might be explained by the fact that in this low-risk subgroup, letrozole adverse events possibly leading to mortality outweigh the benefit of letrozole on breast cancer-related mortality. However, this does not explain why we see the same trend for longer therapy on distant metastasis-free interval. Further evaluations in larger analyses from collaborative groups, in the setting of a meta-analysis, are required to validate this effect.

A limitation of this trial is that the analyses were performed in a subgroup of the original trial population, and this subgroup analysis was therefore not powered to detect small differences and might have suffered from multiple-testing error. Furthermore, in view of the design of the IDEAL study (having two treatment arms and no placebo arm), it was not possible to investigate the value of extended therapy versus no extended therapy.

In conclusion, the results of the current exploratory analysis in IDEAL patients suggest that longer (vs. shorter) extended endocrine therapy might be of value for node-positive patients, and in particular for those who were treated with tamoxifen followed by an AI for the first 5 years, which was not observed in the AI monotherapy subgroup. For all node-negative patients, there was no beneficial effect of longer therapy, and even a trend toward a worse outcome. Future studies, and future meta-analyses, are warranted to validate these results, and to further identify for which subgroup there is an effect of extended endocrine therapy after optimal endocrine therapy over the first 5 years.
